# Gene editing in small and large animals for translational medicine: a review

**DOI:** 10.1590/1984-3143-AR2023-0089

**Published:** 2024-04-12

**Authors:** Clésio Gomes Mariano, Vanessa Cristina de Oliveira, Carlos Eduardo Ambrósio

**Affiliations:** 1 Departamento de Medicina Veterinária, Faculdade de Zootecnia e Engenharia de Alimentos, Universidade de São Paulo – USP, Pirassununga, SP, Brasil

**Keywords:** animal models, CRISPR, gene editing, translational medicine

## Abstract

The CRISPR/Cas9 system is a simpler and more versatile method compared to other engineered nucleases such as Zinc Finger Nucleases (ZFNs) and Transcription Activator-Like Effector Nucleases (TALENs), and since its discovery, the efficiency of CRISPR-based genome editing has increased to the point that multiple and different types of edits can be made simultaneously. These advances in gene editing have revolutionized biotechnology by enabling precise genome editing with greater simplicity and efficacy than ever before. This tool has been successfully applied to a wide range of animal species, including cattle, pigs, dogs, and other small animals. Engineered nucleases cut the genome at specific target positions, triggering the cell's mechanisms to repair the damage and introduce a mutation to a specific genomic site. This review discusses novel genome-based CRISPR/Cas9 editing tools, methods developed to improve efficiency and specificity, the use of gene-editing on animal models and translational medicine, and the main challenges and limitations of CRISPR-based gene-editing approaches.

## Introduction

The discovery of a type of prokaryotic repetitive DNA (Ishino et al., 1987), its recognition as a family (Mojica et al., 2000), the discovery of its palindromic nature, identification of the Cas genes, and the first denomination of CRISPR (Clustered Regularly Interspaced Short Palindromic Repeats)(Jansen et al., 2002), paired with the understanding and posterior confirmation that it was part of a primitive adaptive immune system (Mojica et al., 2005; Bolotin et al., 2005; Barrangou et al., 2007) and its further development as a tool for genome engineering (Jinek et al., 2012; Cong et al., 2013) is revolutionizing biotechnology and has led to the modification of prokaryotic and eukaryotic genomes with much greater simplicity and efficacy than ever before (Knott and Doudna, 2018). CRISPR was first shown to be functional as a genome editing tool in mammalian cells in 2013 (Cong et al., 2013) and then applied to many cell lines and species, including cattle (Ikeda et al., 2017), pigs (Whitworth et al., 2016; Yang et al., 2018), pets (Amoasii et al., 2018) and other small animals and large animals (Sui et al., 2018; Lin et al., 2022). This technology enables modifications that result in enhancements in animal production traits, animal health and welfare, as well as the creation of more refined animal models for studying human diseases. It also allows for the production of pharmaceutical proteins and the investigation of gene function (Wang and Doudna, 2023).

Precise genome editing relies on engineered nucleases, such as Zinc Finger Nucleases (ZFNs), Transcription Activator-Like Effector Nucleases (TALENs), and more recently CRISPR, to target specific sites in the genome and introduce mutations (McMahon et al., 2011; Perisse et al., 2021). Among these methods, CRISPR/Cas9 has gained prominence due to its streamlined and flexible approach, eliminating the need for constructing custom-engineered proteins for each target. Additionally, the efficiency of CRISPR/Cas9 has significantly improved, enabling multiple edits simultaneously (Georges et al., 2019). Consequently, it has become the preferred method for precise genomic modifications in animals. The field of CRISPR/Cas9 editing tools continues to expand, with the development of novel genome-based methods (Chen and Liu, 2023; Wang and Doudna, 2023). This review will focus on discussing these methods, with an emphasis on enhancements to efficiency and specificity of CRISPR-based tools like nCas9 and dCas9. It will also explore approaches for gene regulation, base editing, and epigenetic modifications. Furthermore, the application of gene-editing technology in biomedicine, particularly in animal models and translational medicine, will be examined.

## Animal models and translational medicine

Animal models have been used for experimental surgery and medical research for over a millennium, with a history that dates back to the earliest days of human civilization. In fact, the first textbooks on anatomy were based on dissection of pigs and apes, rather than human cadavers (Ericsson et al., 2013). Since then, the use of animal models has played a significant role in many of the greatest scientific discoveries in history, including William Harvey's work on circulation (Aird, 2011) and Louis Pasteur's groundbreaking research in microbiology (Lobanovska and Pilla, 2017). In modern times, animal models continue to be a crucial tool in translational medicine, with applications ranging from cancer research to neurodevelopmental disorders, such as autism (Silverman et al., 2012). For example, xenografting, a technique in which human cancer tissue is transplanted into immunosuppressed mice, allows researchers to study the development of cancer *in vivo* (Abdolahi et al., 2022). One of the most significant recent developments in animal modeling is the production of humanized mouse models. These models involve the engraftment of human hematopoietic stem cells into mice with targeted mutations in genes to knock out the immune response. This creates a mouse model with a human immune system, which can be used to study various research fields, including immune, infectious, and oncology research. These models are considered central to recent and future advances in translational research, including pharmaceutical development and personalized medicine (Fujiwara, 2018). However, despite the benefits of animal models in translational medicine, they also have limitations that must be taken into account. One of the most significant limitations is the natural physiological and pathological differences between animals and humans. For instance, young and healthy animals used for research always carry a risk of selection bias. To overcome these limitations, interdisciplinary approaches are required, involving work at the genetic, molecular, cellular, and clinical scale to understand the link between these elements within animals and humans (Robinson et al., 2019).

## Gene editing

**Figure 1:**
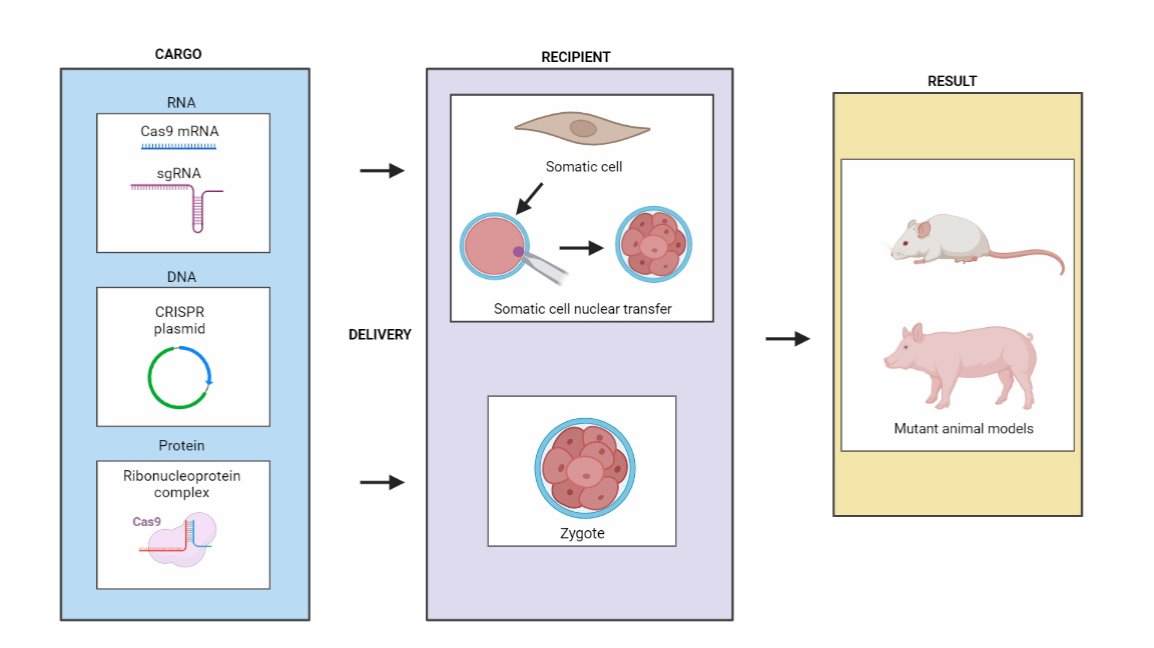
Schematic summary of different CRISPR approaches for generation of edited animal models.

Numerous comprehensive reviews have been published on the topic of gene-editing technology and gene-edited (GE) animal models, including those by Roth and Tuggle (2015), Kalds et al. (2019, 2020), McFarlane et al. (2019), Lee et al. (2020), Menchaca et al. (2020), Navarro-Serna et al. (2020), Maynard et al. (2021), Perisse et al. (2021), Hay et al. (2022), Lin et al. (2022) and Camargo et al. (2023). In this article, we aim to provide an overview of key developments and recent advancements in the CRISPR/Cas9 field, along with a review of relevant literature focused on the generation of edited cell lines and animals (Figure 1) with specific biomedical and veterinary applications, building upon the existing literature.

## Zinc fingers (ZFNs) and TALENs

In 2001, Bibikova et al. (2001) developed chimeric nucleases known as zinc finger nucleases (ZFNs) with the ability to target and disrupt specific DNA sequences. Zinc fingers are small protein motifs that bind to DNA and recognize a 3-base pair (bp) sequence. These motifs were combined with the genetically modified restriction enzyme FokI to create a programmable nuclease that could identify target sequence sites. Two zinc finger modules bind to DNA at sites that oppose each other with the FokI enzyme in the middle to form a homodimer complex. Once homo-dimerization is established, the nuclease breaks both strands of DNA and randomly inserts mutations (Adli, 2018). By changing the residues in a single zinc finger, the target site can be designed to recognize many different DNA triplets (Carroll, 2017). Although ZFNs are highly specific to DNA sequences, they have several major drawbacks, including a time-consuming design process and limited potential targets in the genome, making them unsuitable for many gene-editing projects (Qomi et al., 2019; Hirakawa et al., 2020).

In 2009, a new generation of nucleases emerged, known as transcription activator-like effectors (TALEs), which were originally found in plant pathogenic bacteria from the genus *Xanthomonas* (Boch et al., 2009). TALEs are DNA-binding domains containing repeat motifs of 33-35 amino acids that identify each nucleotide, with their site-specificity determined by two hypervariable amino acids known as repeat-variable di-residues (Gaj et al., 2013; Joung and Sander, 2013). Similar to ZFNs, TALEs have been engineered to fuse with the DNA-cutting domain of the FokI nuclease, creating TALENs as a gene-editing tool (Thakore and Gersbach, 2016; Adli, 2018). The main difference between ZFNs and TALENs is the number of nucleotides recognized by the protein domains, with TALENs recognizing one bp, making them more site-specific and less likely to cause off-target cleavage (Khan, 2019; Hirakawa et al., 2020; Bhardwaj and Nain, 2021; Perisse et al., 2021).

## CRISPR

The CRISPR system is based on RNA sequences originally discovered by Ishino et al. (1987) in *Escherichia coli*, and named Clustered Regularly Interspaced Short Palindromic Repeats (CRISPR) by Mojica et al. (2000) and Jansen et al. (2002). The CRISPR locus found in some prokaryotes is a primitive acquired immune system that defends against foreign DNA, such as bacteriophage (Humphrey and Kasinski, 2015). The CRISPR system consists of two phases: the immunization phase and the immunity phase. In the immunization phase, the Cas1 and Cas2 endonucleases recognize the viral genome, cuts it, and insert fragments into the bacterial genome as repeat-spacer units. During a subsequent viral invasion, the bacteria produce precursor-CRISPR RNA (pre-crRNA), which binds to the Cas9 endonuclease and trans-activating crRNA (tracrRNA) to form the crRNA-Cas9-tracrRNA complex. The complex is then degraded by RNase III, resulting in small crRNA guides for targeting exogenous DNA and promoting the DSB of invading viral DNA (Marraffini, 2015; Qomi et al., 2019; Perisse et al., 2021).

The CRISPR/Cas9 system consists of the Cas9 endonuclease bound to a tracrRNA/crRNA duplex, where the crRNA region contains 20 customizable nucleotides that form the guide RNA (gRNA), and the tracrRNA consists of 14 nucleotides anti-repeat region and three loops (Mei et al., 2016). The duplex RNA guides the Cas9 to the specific sequence on the target DNA where the gRNA aligns against the complementary sequence. The helicase domain of Cas9 opens the double strands while the nuclease sites (RuvC and HNH) perform the DSB of the DNA. The designed target sequence must be located upstream of a protospacer adjacent motif (PAM) – 5’-NGG-3’ where N can be any of the four known DNA nucleotides to be recognized by the Cas9 nuclease (Yang, 2015). The Cas9 starts the target site-searching process by probing a suitable PAM sequence before matching the gRNA complementary to the DNA. The Cas9 triggers the DSB only after a precise complementarity between the gRNA and the target DNA has been reached, which provides the energy to the enzyme to break the DNA (Jiang and Doudna, 2017; Hirakawa et al., 2020). The CRISPR/Cas9 system has been extensively modified to increase its efficiency and specificity, making it a powerful tool for genome editing (Sander and Joung, 2014; Wang and Doudna, 2023). Although Jinek et al. (2012) were the first to describe CRISPR as a gene editing tool, which led to a Nobel Prize for chemistry in 2020 to Dr. Jennifer Doudna and Dr. Emmanuelle Charpentier, a group led by Dr. Feng Zhang was the first to actually use it in mammalian cells (Cong et al., 2013). A summary of the differences of CRISPR and its main variations is available at the end of this section (Table 1).

**Table 1:** CRISPR and its main formats.

**CRISPR Variation**	**Main Characteristics**	**Enzyme**
Traditional CRISPR (CRISPR/Cas9)	Uses Cas9 enzyme to cut both strands of DNA at specific locations guided by a guide RNA (gRNA). Allows for gene disruption, insertion, or modification by inducing double-strand breaks (DSBs) in the DNA.	Cas9
CRISPRi (CRISPR Interference)	Utilizes a modified Cas9 enzyme (dCas9) to block gene expression without altering DNA sequence. dCas9 is guided to specific genomic locations by gRNA, where it interferes with transcription by steric hindrance or recruitment of repressive proteins.	dCas9 (Catalytically inactive Cas9)
CRISPRa (CRISPR Activation)	Employs a modified Cas9 enzyme (dCas9) to activate gene expression without altering DNA sequence. dCas9 is guided to target genes by gRNA, where it recruits transcriptional activators to enhance gene expression.	dCas9 (Catalytically inactive Cas9)
Prime Editing	Introduces precise edits to the DNA without requiring DSBs. Utilizes a fusion protein of a Cas9 nickase and a reverse transcriptase enzyme guided by a prime editing guide RNA (pegRNA) to make specific changes to the DNA sequence.	Cas9 nickase
Base Editing	Allows for the conversion of one DNA base pair into another without inducing DSBs. Utilizes a fusion protein of a Cas9 nickase and a base editing enzyme (e.g., cytosine or adenine deaminase) guided by a gRNA to directly convert one base pair to another.	Cas9 nickase
Cas9 Nickase	A modified version of the Cas9 enzyme that cuts only one strand of the DNA double helix. Reduces off-target effects and allows for more precise editing by inducing single-strand breaks (nicks) instead of DSBs.	Cas9 nickase

## Main CRISPR variations

### Cas9 nickase (nCas9)

The efficiency and specificity of gene-editing using Cas9 endonuclease have been improved through the engineering of a modified version known as Cas9 nickase (nCas9) (Ran et al., 2013). The nCas9 creates a single-strand DNA break through one functional domain while the other domain is inactivated. By using two gRNAs and performing “double nicking,” the chances of off-target mutation events are reduced without affecting on-target efficacy (Cho et al., 2014; Adli, 2018). While the CRISPR/Cas9 system can tolerate up to six nucleotide mismatches, undesirable off-target mutations can still occur and reduce cell viability. However, the use of double nicking has been shown to decrease off-target activity by 50 to 1,500-fold in cell lines (Zhang et al., 2015; Harrison and Hart, 2018).

### CRISPRi and CRISPRa (dCas9)

The CRISPR/Cas9 system has been modified to produce the nuclease-null deactivated Cas9 or “dead Cas9” (dCas9), which retains the ability to target specific sequences without causing any damage to the DNA (Qi et al., 2013). The dCas9 can be combined with regulatory factors to turn genes on or off and adjust their level of activity. For example, the Cas9 protein fused with Kruppel-associated box (KRAB) promotes gene repression while the enzyme fused with VP16 or VP64 activates gene expression (Gilbert et al., 2013; Lawhorn et al., 2014; Gao et al., 2014). This allows for the precise placement of modifications that regulate gene expression and DNA dynamics, offering the potential to correct epigenetic disorders. CRISPR interference (CRISPRi) uses dCas9 to reversibly turn off genes by targeting, but not cutting a specific site and epigenetically modifying the gene, inhibiting transcription. Conversely, CRISPR-mediated activation (CRISPRa) promotes gene transcription. With these tools, epigenetic marks in different cells can be precisely modified to regulate the effects on gene expression, providing new possibilities for research into everything from tumor growth to brain activity (Dominguez et al., 2016; Mei et al., 2016).

## Base editing and prime editing

Base editors (BE) are a breakthrough in gene editing due to their ability to perform precise point mutations without the need for a double-strand break (DSB). They consist of a programmable DNA-binding protein, such as a catalytically impaired Cas nuclease (Cas9 nickase) fused to a deaminase enzyme, which converts one base to another. Guide RNA targets the base editor to bind a matching sequence within genomic DNA. Cytosine base editors (CBEs) and adenine base editors (ABEs) catalyze C•G-to-T•A and A•T-to-G•C changes, respectively. C•G-to-G•C base editors (CGBEs) are similar to CBEs but stimulate the replacement of the deaminated cytosine base with guanine. Base editors are more efficient than Cas nucleases, exhibit fewer indel byproducts and show fewer undesired consequences of double-strand breaks (DSBs). However, base-editing activity is restricted by the targeting scope of the Cas domain, which requires the presence of a protospacer adjacent motif (PAM) sequence. Also, some base editors can induce off-target mutations in DNA and RNA. At present, base editors are limited in their ability to produce only six out of the 12 potential types of point mutations, leaving a wide range of DNA edits inaccessible. The initial base editor, BE1, involved fusing CRISPR/dCas9 with a cytidine deaminase to directly convert cytidine to uridine. BE1 specifically targeted nucleotides positioned within 4–8 base pairs of the PAM sequence (Komor et al., 2016). However, BE1 was not highly efficient in converting the U.G pair to a T.G pair. To address this limitation, Dr. Liu and colleagues developed a novel version called BE2, which incorporated an uracil DNA glycosylase inhibitor (UGI), a small protein derived from the bacteriophage primer binding site (PBS), fused to the C-terminus of BE1 (Rees and Liu, 2018). BE2 significantly improved the efficiency of the conversion, resulting in a three-fold increase compared to BE1. To further enhance the efficiency of base editing, the researchers restored the catalytic histidine residue at position 840 in the Cas9 HNH domain of BE2, creating the third-generation base editor, BE3. BE3 proved to be significantly more effective, achieving up to 37% conversion of C-to-T across the entire DNA (Komor et al., 2017; Koblan et al., 2018). Numerous other variants of cytidine base editors have been developed, leading to further improvements in C-to-T editing. Another notable advancement is the adenosine base editor (ABE), a new generation of base editor that can convert A.T base pairs to G.C nucleotides and has the potential to reverse pathogenic mutations (Gaudelli et al., 2017). These optimized base editors have been successfully utilized in various organisms, including mouse, rabbit, pig, and human cells (Kim et al., 2017; Zafra et al., 2018; Xie et al., 2019; Liu et al., 2020; Perisse et al., 2021).

Prime editing is a gene-editing technology that addresses the need for precision and versatility, allowing for targeted installation of all types of DNA substitution, small insertions, and small deletions in living cell genomes without the formation of direct double-strand breaks (Chen and Liu, 2023). Unlike traditional CRISPR/Cas9-based methods, prime editing uses a catalytically impaired Cas9 nickase (H840A) fused with a reverse transcriptase (RT-nCas9) and a 3’-extended guide RNA (pegRNA) to introduce precise point mutations in the genome (Anzalone et al., 2019, 2020). The pegRNA molecule contains a target sequence at its 5’ end, which recognizes the DNA target site, and a long 3’ end that extends to interact with the opposite strand of the target sequence. The RT-nCas9 nickase then breaks the single-strand DNA via the RuvC nuclease domain. Once the DNA strand is broken, the tip of the 3’ end of the pegRNA, which contains a primer binding site (PBS), aligns against the broken DNA strand. The RT-nCas9 uses the pegRNA template containing the modification site upstream to the PBS to synthesize a brand new sequence. The principal advantage of prime editing lies in its ability to encode both the site to be targeted and the nature of the repair within a single molecule, the pegRNA. This allows for more efficient and precise edits, as well as the ability to insert point mutations without the use of a donor DNA template for the homology-directed repair (HDR) pathway. Additionally, the incorporation rate can be further enhanced using an additional gRNA, which makes a nick on the opposite strand, boosting DNA repair with the 3′-flap sequence, albeit with a decrease in precision (strategy referred to as PE3/PE3b). Anzalone et al. (2019), used the PE3 strategy to demonstrate many classes of precise edits, including the programming of deletions ranging from 5 to 80 bp with high efficiency (52-78%) and modest precision (11% rate of unintended indels on average) (Anzalone et al., 2019). One of the latest prime editing improvements consists of a split PE (sPE) that separates the Cas9 nickase (nCas9) from the reverse transcriptase (RT), resulting in comparable precision editing rates to the unsplit parental PE3 and no increase in the production of insertion-deletion (indel) byproducts, with the advantage of reducing the construct complexity and facilitating delivery. Administering sPE to the liver of mice via hydrodynamic injection to modify β-catenin led to tumor formation with equivalent efficiency to PE3. Similarly, delivering sPE using two adeno-associated virus (AAV) vectors corrected a disease-causing mutation in a mouse model of type I tyrosinemia (Liu et al., 2022).

## Gene-edited animal models

Gene-edited (GE) animal models play a crucial role in advancing our comprehension of disease mechanisms, as they share significant anatomical and physiological similarities with humans. These models are likely to uncover novel clinically relevant mechanism-based targets for the prevention and treatment of various diseases (Roth and Tuggle, 2015; Polejaeva et al., 2016). Large mammal models have undeniably made substantial contributions to translational medicine by effectively representing the complexity of outbred species. Furthermore, they often exhibit pathogenesis patterns in genetic, metabolic, infectious, and neoplastic diseases that are more akin to those observed in humans compared to mouse model equivalents (Reynolds et al., 2009). Livestock models possess similar organ sizes and functions, rendering them more suitable than mice for numerous biomedical applications. These applications include tissue recovery, serial biopsies, blood sampling, device development, whole-organ manipulations, cloning, and the development of surgical procedures (Reynolds et al., 2009; Lin et al., 2022). The current availability of genome sequences and efficient gene-editing techniques has further enhanced the accessibility of GE animal models, facilitating their widespread utilization in research and experimentation (Maynard et al., 2021; Perisse et al., 2021; Lin et al., 2022). A summary of the edited cell lines and animal models shown in this review is available at the end of this section (Table 2).

**Table 2:** CRISPR-mediated approaches for generating disease animal models and correcting genetic diseases.

**Species**	**Genes**	**Purpose**	**Approach**	**References**
Goat	*MSTN, PrP, NUP, BLG*	Creating knockout models for diseases	CRISPR/Cas9-mediated knockout	Ni et al. (2014)
Sheep	*CFTR*	Creating model for cystic fibrosis	CRISPR/Cas9-mediated knockout	Fan et al. (2018)
Bovine	*IARS*	Repairing recessive mutation	CRISPR/Cas9-mediated knock-in	Ikeda et al. (2017)
Bovine	*TFAM*	*In vitro* model for mitochondrial diseases	CRISPR/Cas9-mediated knockout	Oliveira et al. (2019, 2020)
Pig	*HTT*	Creating model for Huntington's disease	CRISPR/Cas9-mediated knock-in	Yan et al. (2018)
Pig	*IAPP*	Creating model for type 2 diabetes mellitus	CRISPR/Cas9-mediated knock-in	Zou et al. (2019)
Pig	*CD163*	Creating model for PRRSV	CRISPR/Cas9-mediated knockout	Whitworth et al. (2014), Yang et al. (2018), Tanihara et al. (2021)
Pig	*APN*	Creating model for TGEV and PDCoV	CRISPR/Cas9-mediated knockout	Whitworth et al. (2019), Xu et al. (2020)
Pig	*DMD, TYR, LMNA, RAG1, RAG2, IL2RG*	Creating pigs with gene mutations	Cytosine base editors (CBEs)	Xie et al. (2019)
Rabbit	*DMD*	Creating model for Duchenne Muscular Dystrophy	CRISPR/Cas9-mediated knockout	Sui et al. (2018)
Dog	*DMD*	Developing therapy for Duchenne Muscular Dystrophy	CRISPR/Cas9-mediated knockout	Amoasii et al. (2018)
Cat	*HAP2*	Creating mutant parasite for T. gondii vaccine	CRISPR/Cas9	Ramakrishnan et al. (2019)
Cat	Type I interferon signaling genes	Studying FIPV and vaccine production	CRISPR/Cas9	Mettelman et al. (2019)
Cat	FeLV	Reducing FeLV viral load	CRISPR/Cas9	Helfer-Hungerbuehler et al. (2021)
Zebrafish	Many genes	Creating knock-in models for diseases	CRISPR/Cas9-mediated knock-in	Kalueff et al. (2014), Bellipanni et al. (2016), Gore et al. (2018), Outtandy et al. (2019), Wang et al. (2021), Katoch and Patial (2021), Hoareau et al. (2022)

## Small and large ruminants

Sheep and goats have been used as models in biomedical research due to their advantages in size and physiology when compared to more common experimental models such as rodents (Lin et al., 2022). The first goat knockout model created by CRISPR/Cas9 was reported in 2014 by Ni et al. (2014). In this study, authors first disrupted four genes (*MSTN, PrP, NUP* and *BLG*) in primary fibroblasts and then proceeded to produce biallelic mutated goats with somatic cell nuclear transfer (SCNT), showing that CRISPR/Cas9-mediated gene knockout followed by SCNT is an efficient approach to create large mutated animal models. In 2018, Fan et al. (2018) successfully created the first sheep model for human cystic fibrosis (CF). They achieved this by utilizing CRISPR/Cas9 to disrupt the cystic fibrosis transmembrane conductance regulator (*CFTR*) gene. The resulting newborn *CFTR*−/- sheep exhibited severe disease symptoms consistent with CF. These symptoms included pancreatic fibrosis, intestinal obstruction, as well as significant liver and gallbladder abnormalities that mirror CF-related liver disease in humans. Aside for creating animal models for human diseases, gene-editing techniques have shown promise in effectively correcting disease-causing mutations. In a notable study, Ikeda et al. (2017) successfully repaired a recessive mutation responsible for isoleucyl-tRNA synthetase (IARS) syndrome in Japanese Black cattle. Over the course of more than six decades, selective breeding aimed at achieving high meat quality and distinct marbling in these cattle inadvertently led to the accumulation of recessive mutations associated with genetic disorders. Specifically, a substitution (c.235G > C, p.Val79Leu) in the *IARS* gene resulted in a 38% decrease in the aminoacylation activity of the IARS protein, leading to impaired protein synthesis. Calves homozygous for this mutant allele displayed neonatal weakness and intrauterine growth retardation.

CRISPR-based approaches can also be used to create new *in vitro* disease models. Recently, Brazilian researchers reported the creation (Oliveira et al., 2019) and characterization (Oliveira et al., 2020) of an *in vitro* bovine model for mitochondrial diseases by monoallelic knockout of the *TFAM* gene in primary bovine fibroblasts, which resulted in a decreased level of mitochondrial DNA copy number. This *TFAM* mutated phenotype is associated with some human diseases, including Moyamoya disease (Key et al., 2020) and Perrault Syndrome (Tucker et al., 2020).

## Pigs

The porcine animal model offers numerous advantages compared to other models due to its short reproductive cycle, early sexual maturity, and ability to produce high-numbered litters (Zou et al., 2019). These characteristics greatly facilitate the establishment of GE lineages. Additionally, pigs possess anatomical, biochemical, and physiological features that closely resemble those of humans (Roura et al., 2016), making them highly reliable models for biomedical research (Meurens et al., 2012).

In 2018, researchers achieved a significant milestone by creating a pig model of Huntington's disease (HD) that exhibited selective neurodegeneration, resembling the condition observed in HD patients. This breakthrough was accomplished through a combination of CRISPR/Cas9 knock-in and somatic cell nuclear transfer, resulting in the insertion of a large CAG repeat in the *HTT* gene, which enabled the pigs to naturally produce the mutant huntingtin protein (HTT) associated with HD. Subsequent generations (F1 and F2) of these genetically modified pigs were successfully bred, demonstrating consistent movement and behavioral abnormalities, as well as early mortality, which could be inherited across generations. Notably, the brains of these HD pigs exhibited significant and selective degeneration of striatal medium spiny neurons (Yan et al., 2018). In 2019, researchers obtained *IAPP* gene-humanized miniature pigs via CRISPR/Cas9 and somatic cell nuclear transfer as a model for studying the pathogenesis and related complications of type 2 diabetes mellitus (Zou et al., 2019).

Porcine reproductive and respiratory syndrome virus (PRRSV) poses a significant threat to swine production globally, resulting in severe economic losses. Highly pathogenic PRRSV (HP-PRRSV), derived from a genotype 2 PRRSV, is even more virulent, exacerbating the economic impact. Several research groups have successfully utilized CRISPR/Cas9 gene editing to generate CD163 knockout (KO) pigs (Whitworth et al., 2014; Yang et al., 2018; Tanihara et al., 2021). Experimental infection with either the NVSL 97-7895 PRRSV virulent virus isolate (Whitworth et al., 2016) or the HP-PRRSV strain (Yang et al., 2018) demonstrated that CD163 KO pigs are completely resistant to viral infection. These pigs exhibited the absence of viremia, antibody response, high fever, or any other clinical signs associated with PRRS, while wild-type controls displayed typical signs of PRRSV infection (Whitworth et al., 2016; Yang et al., 2018). Furthermore, recent studies by Whitworth et al. (2019) revealed that pigs lacking aminopeptidase N (APN) are fully resistant to transmissible gastroenteritis virus (TGEV) but not porcine epidemic diarrhea virus (PEDV). In 2019, Xie et al. demonstrated the generation of pigs with single or multiple gene point mutations using cytosine base editors (CBEs), employing either embryo injection or nuclear transfer techniques. The disrupted genes included *DMD, TYR*, and *LMNA*, as well as *RAG1, RAG2*, and *IL2RG*, at both embryonic and cellular levels. The CBEs were also effective in introducing multiple premature stop codons in genes with multiple copies, such as the pol gene of porcine endogenous retrovirus (Xie et al., 2019).

Moreover, studies have shown that porcine alveolar macrophages derived from APN-deficient pigs are resistant to porcine deltacoronavirus (PDCoV). However, lung fibroblast-like cells derived from these animals supported a high level of PDCoV infection, suggesting that APN is not an essential receptor for PDCoV (Stoian et al., 2020). Also, double-gene-knockout (DKO) pigs, with knockouts for both CD163 and pAPN receptor proteins, have been reported to be completely resistant to genotype 2 PRRSV and TGEV (Xu et al., 2020).

## Rodents

Duchenne Muscular Dystrophy (DMD) is a fatal disorder caused by mutations in the dystrophin gene characterized by progressive muscular weakening. Different mutations, such as large deletions and duplications, point mutations and small indels in one or more of the 79 exons present in the *DMD* gene can cause this disease. Due to its relatively high incidence – ~1 in 3500 male births – (Mendell and Lloyd-Puryear, 2013) and currently no effective therapeutic treatment available, researchers are always in need of animal models for DMD. Many preclinical studies were carried out in *Mdx* mice, a widely used animal model presenting the dystrophic phenotype associated with DMD (Bulfield et al., 1984; Chapman et al., 1989; Sicinski et al., 1989), however, studies with this model aren’t easily translated to effective therapies, mostly because the *Mdx* mice phenotype is much milder than DMD’s (Chamberlain et al., 2007). More faithful animal models for DMD – showing severe muscular dystrophy, respiratory distress, and elevated serum creatine kinase – include pigs (Klymiuk et al., 2013) and dogs (Sharp et al., 1992; Baltzer et al., 2007; Smith et al., 2011). As a drawback, generating and maintaining these large animals is a very laborious and costly task, acting as a barrier for many research teams. To facilitate research and tackle these problems a rabbit DMD model was generated by targetting exon 51 of the *DMD* locus (Sui et al., 2018). These *DMD* KO rabbits possessed many hallmarks of the disease, including cardiomyopathy and a high incidence of early-onset death, facilitating basic research and translational studies as a way of developing therapeutic strategies against Duchenne muscular dystrophy.

## Pets

An essential step for successful clinical translation of gene-editing approaches is demonstrating they are safe to use and effective when applied in large mammals. The deltaE50-MD canine model of Duchenne Muscular Dystrophy (DMD) possesses a loss of exon 50 of the *DMD* gene and is clinically very similar to the human disease, displaying its common pathological features – weakening and atrophy of the muscles, cardiomyopathy, and fibrosis (Walmsley et al., 2010). A preliminary study published in 2018 showed up to 90% of dystrophin normalization after 8 weeks of systemic injection in dogs of a CRISPR construction delivered by adeno-associated viruses and targeting a region adjacent to the *DMD*’s exon 51 splice acceptor site. This construction was intended to allow skipping of the exon 51, thus correcting the DMD reading frame and restoring protein expression and function (Amoasii et al., 2018), a fundamental first step toward developing safe therapies for DMD.

Aside from disease animal models, gene-editing techniques can potentially produce different therapeutic solutions, like attenuated live vaccines. This is the case for an experimental vaccine-like approach aimed at tackling felids shedding of *Toxoplasma gondii* oocysts in the environment, a key stage in the life cycle of this zoonotic parasite. After identifying genes coding for micro and macrogamete-specific proteins with putatively important roles in *T. gondii* fertilization and oocyst wall-formation, researchers were able to KO by CRISPR/Cas9 one of the fertilization factors, HAP2, thus creating a mutant parasite incapable of forming infectious sporozoites in *T. gondii* oocytes (Ramakrishnan et al., 2019). Infection with these HAP2 KO parasites, incapable of completing fertilization and undergoing meiosis and only shedding a small number of aberrant putatively non-infectious oocysts acts as a *de facto* immunization for cats against infectious wild-type *T. gondii,* completely blocking the transmission of this parasite. In this same approach (CRISPR systems used to potentially produce vaccines), Mettelman et al. (2019) utilized CRISPR/Cas technology to disrupt type I interferon signaling in two feline cell lines, AK-D and Fcwf-4 CU, and assessed the replication kinetics of serotype I feline infectious peritonitis virus (FIPV) within these as a first step to potentially use the edited cell lines to isolate new clinical FIPV strains and propagate the ones that are candidate for vaccine production.

Also, an *in vitro* study published in 2021 evaluated the efficiency of a CRISPR/Cas9 system on different sites within the feline leukemia virus (FeLV) provirus, with the intention of reducing this retrovirus viral load and thus achieving regressive infection and better clinical outcome. To accomplish this, nine natural AAV serotypes, two AAV hybrid strains, and Anc80L65 (an *in silico* predicted AAV ancestor) were utilized to infect various feline cell lines and primary cells. The research team confirmed the introduction of double-strand breaks using the CRISPR/Cas9 system on 12 selected FeLV provirus sites via T7 endonuclease 1 (T7E1) and Tracking of Indels by Decomposition (TIDE) analysis. Additionally, subsequent transduction experiments utilizing AAV-DJ confirmed indel formation and demonstrated a considerable reduction in FeLV p27 antigen for some targets (Helfer-Hungerbuehler et al., 2021).

## Fish

Aside for mammals, one of the most common animal models used for translational medicine is fish, particularly zebrafish (*Danio rerio*), due to its genetic and experimental accessibility and the transparency of its embryos and larvae. As of 2023, there already are zebrafish models for many human pathologies, such as complex brain disorders (Kalueff et al., 2014), genetic chaperonopathies (Bellipanni et al., 2016), hematopoietic disease (Gore et al., 2018), kidney disease (Outtandy et al., 2019), fibrotic disease (Wang et al., 2021), liver diseases (Katoch and Patial, 2021), vascular pathologies (Hoareau et al., 2022), and many more. In the last decade, the main approach for generating disease models in zebrafish has been CRISPR/Cas9-based knock-in (via homologous directed repair and also insertions by non-homologous end joining) (Albadri et al., 2017).

## Xenotransplantation

Xenotransplantation presents a promising solution to the scarcity of donor organs in allotransplantation, considering that thousands of patients die annually while awaiting transplantation. Recent advancements in interspecies chimerism enabled by CRISPR/Cas9 (Wu et al., 2017), and xenografts, including successful long-term survival of pig organ grafts in non-human primates (Mohiuddin et al., 2016; Längin et al., 2018; Kim et al., 2019; Ma et al., 2022; Mohiuddin et al., 2022), have sparked renewed enthusiasm for this approach. The pioneering pig-to-human heart transplant in 2022 (Griffith et al., 2022), alongside encouraging data from experiments using pig kidneys (Montgomery et al., 2022), underscores the potential of xenotransplantation to address organ shortages. Crucial to these advancements are genetic modifications enabled by gene-editing techniques, mainly CRISPR–Cas9, which allow for the modification of animal organs more akin to human physiology, reducing rejection risks (Ryczek et al., 2021). Utilizing edited animals offers a dependable supply of quality-controlled organs, optimizing size, structure, and function while mitigating infectious risks. Xenotransplantation could broaden access to transplantation, particularly for those at early stages of organ failure, improving outcomes and quality of life (Sykes and Sachs, 2022). The pig, due to its size, availability, breeding characteristics, and physiological likeness to humans, has emerged as the preferred source animal for xenografts (Xi et al., 2023).

## Gene-editing challenges and limitations

**Figure 2:**
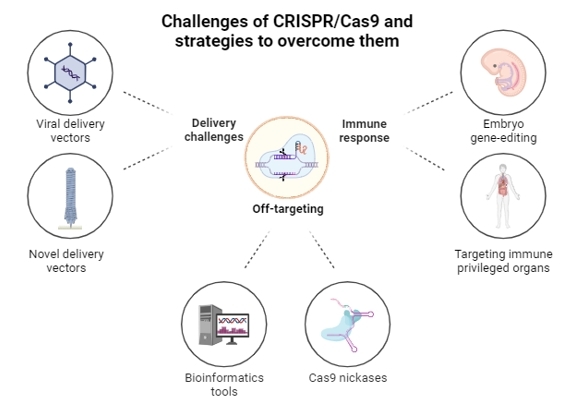
Schematic summary of three of the biggest challenges that CRISPR-based technologies face and some strategies to overcome them. Adapted from Rasul et al. (2022).

CRISPR/Cas9 and its variations, even though are the most widely used gene-editing set of tools from basic to applied research, still face challenges and limitations. There are many reviews addressing CRISPR challenges from different perspectives, such as environmental and health-related safety concerns (Pineda et al., 2019), *in vitro* systems (Ebrahimi and Hashemi, 2020), *in vitro* and *in vivo* delivery (Lino et al., 2018), translation to therapeutic modalities (Tay et al., 2020), and cancer therapy (Rasul et al., 2022). Here, we will discuss four of the most common challenges that scientists face when designing and developing CRISPR-based approaches and some strategies to overcome them (Figure 2).

Possibly the most discussed CRISPR limitation due to its high impact in gene-editing is off-targeting. The RNA guiding system that directs the Cas proteins to the desired *loci* have been shown to still bind to DNA with up to 3-5 mismatches, thus potentially causing off-target DSBs (Ran et al., 2013). By designing better gRNAs with the help of bioinformatics tools researchers are able to reduce off-target effects and screen possible off-target *loci* after the editing is completed. Regarding the structure of the gRNAs, studies have shown that reducing their size to less than 20 nucleotides significantly lowers off-targeting while maintaining editing efficiency (Fu et al., 2014; Chung et al., 2020). Another valid approach to reduce possible off-targets is to use Cas9 nickase (a Cas9 with one of its nuclease domains mutated to be inactive). Because Cas9 nickases are usually employed with double adjacent gRNAs instead of just one their specificity is higher than regular Cas9 approaches (Shen et al., 2014). Also, using double nicking can still cause gene knockouts that require DSBs while having the advantage of the higher specificity and less off-targeting of the Cas9 nickases (Ran et al., 2013).

Another common CRISPR challenge, especially when editing *in vivo* targets, is the immune response of the host organism. This immune response can be triggered by the vectors employed to carry the CRISPR construct (like plasmids and adeno-associated viruses) and also to the Cas9 itself. The most studied and used Cas9 derives from *Streptococcus pyogenes,* a common mammal pathogenic agent, which causes the immune system to recognize the SpCas9 as an antigen and act upon it, which leads to degradations and loss of function (Crudele and Chamberlain, 2018; Charlesworth et al., 2019). To overcome this challenge in *in vivo* approaches scientists can opt to perform the editing early during development, when the immune system isn’t fully functional (Kanellopoulos-Langevin et al., 2003; Kotagama et al., 2019) and also in the so-called immune-privileged organs, organs that naturally have a lower immune activity, such as the brain (Castellani et al., 2023), the eyes (Benhar et al., 2012), and the testicles (Zhao et al., 2014).

One of the biggest challenges when generating GE animal models is genetic mosaicism, which is characterized by the presence of multiple genotypes within a single individual. Mosaicism can arise through various natural mechanisms (Taylor et al., 2014) or manipulative processes such as genome editing (Yen et al., 2014). CRISPR-mediated gene editing in embryos often leads to genetic mosaicism in founders, particularly in the generation of knockout and transgenic animal models (Mehravar et al., 2019). In this method, CRISPR/Cas9 components are injected as DNA, RNA, or protein molecules directly into fertilized zygotes, allowing for continuous targeting and cleavage of genes at different embryonic stages, thus resulting in mosaic animals (Yen et al., 2014). The best way to circumvent mosaicism in GE animals is somatic cell nuclear transfer (SCNT), which was pioneered with the birth of Dolly the sheep (Wilmut et al., 1997) and has since been adapted for various livestock species (Perisse et al., 2021) marking a milestone in livestock genetic engineering. This method involves precise genetic modifications in somatic cells, typically fetal fibroblasts, followed by the isolation of single-cell-derived colonies and cell screening to confirm desired genetic alterations. The resultant cells serve as donor cells for SCNT, where the entire animal originates from a single genetically engineered donor nucleus, reducing the risk of mosaicism (Polejaeva and Campbell, 2000). Despite its technical challenges and low term development rate, SCNT remains the primary method for producing genetically engineered livestock (Lamas-Toranzo et al., 2019; Navarro-Serna et al., 2021). Although there are concerns about potential cloning-related epigenetic alterations and the need for F1 generation animals for proper characterization of genetic models, SCNT continues to be a vital tool in livestock genetic engineering, responsible for approximately half of published knockout farm animals (Perisse et al., 2021).

The fourth great challenge that researchers face when employing CRISPR-based approaches is choosing the right delivery method to introduce the desired CRISPR construction into the host organism. There are many delivery methods, each with its advantages and disadvantages. For instance, delivery by plasmids is a very common approach due to its versatility, size capacity and possibility of expressing fluorescent markers alongside the CRISPR construction that facilitate post-transfection screenings. Plasmids are also expressed in the host cells for longer periods than other methods, which enhances editing efficiency but also causes higher off-target effects. Plasmids also may trigger immune responses after delivery, hindering their usefulness in *in vivo* experiments (Glass et al., 2018). A very common delivery method for *in vivo* CRISPR-based editing are adeno-associated viruses (AAVs), due to its non-immunogenicity, high editing efficiency and relatively low off-target effects. The major disadvantage of this delivery approach is the limited cargo capacity that AAVs have (about 4.7 kpb). The SpCas9 alone is ~4.3 kpb in size, which limits the delivery of extra CRISPR components such as gRNAs and donor DNA for gene insertions by homology-directed repair knock-ins (Mout et al., 2017). Splitting the CRISPR constructions in more than one AAV vector and/or utilizing smaller-sized Cas9 are possible ways to overcome the cargo size limitations for AAVs (Ran et al., 2015). Also, new delivery methods that are being developed may help to overcome the delivery challenges for CRISPR-based technologies. One of the most recent and promising delivery methods was developed by Dr. Feng Zhang’s and team at the Broad Institute. In a 2023 publication they show a novel and programmable protein delivery device based on extracellular contractile injection systems (eCISs) from endosymbiotic bacteria. These eCISs are complexes that inject payloads in a target-specific way. They can also carry relatively big cargo, such as SpCas9 protein and zinc finger deaminases. This new delivery method is being highly praised for its versatility and specificity, making it a potential solution for future gene-editing experiments and therapies (Kreitz et al., 2023).

## Final considerations and future perspectives

The discovery and development of CRISPR technology and CRISPR-based tools has revolutionized biotechnology and genomic engineering, making it possible to modify genomes with greater simplicity compared to other gene-editing methods like TALENs and Zinc Fingers. CRISPR has enabled precise genome editing in a wide range of cell lines and species, including mammals like cattle, pigs, and pets, and has led to the generation of more refined animal models of human diseases and investigation of gene function and molecular mechanisms. Although the CRISPR/Cas9 system is already efficient, efforts are being made to further improve its specificity and accuracy, such as through the development of nCas9 and dCas9 methods (Chen and Liu, 2023). In this regard, recently a team from Osaka University published a new approach called NICER that utilizes nCas9 to induce multiple nicks to correct heterozygous mutations by interhomolog homologous recombination that rarely induces off-target alterations (Tomita et al., 2023). Future directions in this field include exploring the use of gene editing for epigenetic modifications, base editing, and gene regulation, as well as expanding its applications in biomedicine and translational medicine. New gene-editing technologies with the advantages of the RNA-guided nucleases but originated from eukaryotes, such as Fanzor (Saito et al., 2023), might take CRISPR’s place as the most used gene-editing technology in the future, but with its enormous potential, CRISPR technology is poised to continue driving major advances in biology and medicine in the years to come (Wang and Doudna, 2023).
